# Red blood cells exposed to cancer cells in culture have altered cytokine profiles and immune function

**DOI:** 10.1038/s41598-020-64319-3

**Published:** 2020-05-07

**Authors:** Elisabeth Karsten, Edmond Breen, Sharon A. McCracken, Stephen Clarke, Benjamin R. Herbert

**Affiliations:** 1 Translational Regenerative Medicine Laboratory, Kolling Institute, Royal North Shore Hospital, Sydney, Australia; 2 Northern Clinical School, Faculty of Medicine, The University of Sydney, Sydney, Australia; 3 Sangui Bio Pty Ltd, Sydney, Australia; 4 Bioinformatic Consulting, Sydney, Australia; 5 Perinatal Research, Kolling Institute, Royal North Shore Hospital, Sydney, Australia; 6 Cancer Services, Northern Sydney Local Health District, St. Leonards, Sydney, Australia

**Keywords:** Cancer, Immunology

## Abstract

It is now accepted that red blood cells (RBCs) from healthy individuals regulate T-cell activity through modulating cytokine interactions, and that stored RBCs or RBCs from inflammatory cohorts are dysfunctional. Our study aimed to investigate how changes in RBCs that have been intentionally modified can affect T-cell activity as a mechanistic test of this modification. Exposure to a cancer cell line in culture was used to alter the cytokine profile of intact RBCs and the effect of these modified RBCs (ccRBCs) on T-cells was evaluated using flow cytometry. We used RBCs from healthy volunteers and quantified cytokines in RBC lysates and conditioned media using Luminex technology. During *in vitro* cancer cell exposure, RBCs sequestered a variety of cytokines including IL-8, bFGF, and VEGF. Although unmodified RBCs (oRBCs) stimulated proliferation of T-cells (Jurkat cells and peripheral blood mononucleated cells), ccRBCs augmented this proliferative response (3.5-fold and 1.9-fold more respectively). Unlike oRBCs, T-cells stimulated with ccRBCs were no longer protected from phytohemagglutinin-P-driven overexpression of GATA-3 and T-bet and these T-cells were induced to secrete a variety of cytokines including IL-17 and MCP-3. This study supports the hypothesis that RBCs are capable of binding and releasing cytokines in blood, and that modification of these cells can then also affect the T-cell response.

## Introduction

Red blood cells (RBCs) are emerging as important regulators of cell function and survival. A small, but important body of literature has demonstrated that the inclusion of RBCs in culture with other cell types can initiate changes in the secretion profile and in the activity of those cells. For example, RBCs can bind RANTES secreted by endothelial cells, and by doing so, regulate the migration of eosinophils^1^. In addition, incubation of T-cells or mixed lymphocyte populations with RBCs can result in a reduction of apoptosis and a promotion of lymphocyte proliferation, in particular proliferation of CD8+ T-cells^2–4^. RBCs have also been demonstrated to stimulate the T-cell release of inflammatory cytokines including TNF-α and IFN-γ in a dose dependent manner^5^. In recent years, this activity has been shown to be dysregulated in inflammatory conditions. RBCs isolated from participants with carotid atherosclerosis differ from RBCs isolated from healthy individuals in that they have an attenuated capacity to protect T-cells from apoptosis^6^. In addition, following hip arthroplasty RBCs were less able to stimulate T-cell proliferation when compared to pre-surgery samples^7^.

Over the past three decades, there has been some interest in the role of the Duffy antigen receptor for chemokines (DARC) on RBCs due to its ability to bind chemokines. Since its discovery, fewer than ten chemokines have been identified as binding partners to this receptor including IL-8, RANTES, and MCP-1, and RBCs have been suggested to act as a sink for these inflammatory molecules^8,9^. A recent publication has since expanded the list of signalling molecules identified in RBC lysates to include 46 cytokines, chemokines, and growth factors. Indeed, 42 of these cytokines were present in RBC lysates at concentrations ≥4-fold higher than plasma per volume of whole blood^10^. In this publication, they demonstrated that the RBCs were also capable of binding these cytokines, however the capacity to bind individual cytokines has not yet been evaluated^10^. It has been suggested that binding of cytokines to RBCs may have downstream effects on signalling. A 2006 report determined that RBCs rapidly bound chemokines that were released from prostate cancer cells through DARC, and that the presence of this receptor was required to attenuate tumour volume and vessel density^11^.

Abnormal peripheral immune activity and chronic inflammation play a significant role in tumour progression which relies on protection from immune cell-driven apoptosis^12^. A549 cells are derived from a human non-small cell lung carcinoma (NSCLC) cell line that has been investigated extensively due to its capacity to promote an anti-inflammatory microenvironment, a process that is in part modulated by tumour-derived cytokine release and ligation of toll-like receptors on the cells^13^. In patients, NSCLC cells typically secrete pro-angiogenic molecules and a variety of cytokines to promote their growth, in fact plasma levels of a number of these signalling molecules can be correlated back to tumour progression and patient outcome^14^. This cytokine release can induce downstream effects on other cells. In this current study we hypothesised that, in addition to the chemokines already known to bind to RBCs, cytokines would also be able to bind to these cells. Furthermore, cell-cell interactions (in this case, with A549 cells) would modify the RBC cytokine profile and subsequently those cell-contact modified RBCs (ccRBCs) would induce a change in the activity of other cells they come in contact with, notably T-cells. Investigation into this may provide some valuable insight into the use of RBCs as a diagnostic tool, and some clue into understanding the immunological processes of NSCLC biology.

## Results

### RBC purity following dextran sedimentation

The purity of the RBCs was calculated by determining the level of white blood cell and platelet depletion from the original whole blood samples. Following dextran sedimentation, 97.2 ± 3.4% white blood cells and 98.2 ± 3.8% platelets were removed from the RBC samples (*n* = 12). These resulting cells will be referred to as RBCs throughout the manuscript.

### RBC cytokine binding

**Figure 1:**
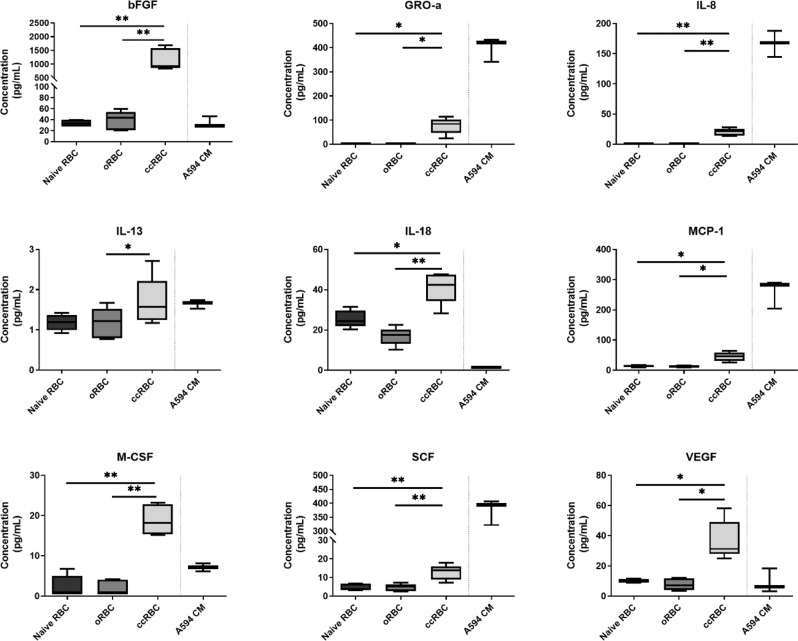
RBC acquisition of cytokines from cancer cells. Summary of cytokines in the lysates of naïve RBCs before incubation, oRBCs and ccRBCs after incubation (3 days, cultured at a ratio of 1:100 A549:RBCs cell number), and conditioned media from A549 cells cultured alone (A549 CM) as measured by Bio-Plex. Lysate data reported as concentration (100 × 10^6^ cells/mL PBS). Data are presented as box and whisker plots with median concentration (n = 5). Data are statistically significantly different if *p < 0.05, **p < 0.01.

Recent research has identified that a range of cytokines are detectable in RBC lysates and that the level of the cytokines in the RBCs increased when these cells were incubated with recombinant cytokines, indicating that they had bound to the cells^10^. In light of these results we sought to investigate if similar increases in cytokine levels could be achieved by incubating RBCs with a cytokine producing cell line. RBCs were incubated with or without a lung cancer cell line (A549 cells) for 3 days to produce ccRBC (co-cultured RBCs) or oRBCs respectively, after which the cytokine profile of the ccRBC and oRBC lysates was determined. The concentration of a representative selection of cytokines is presented in Fig. 1; the data for the remaining cytokines can be found in Supplementary Figs. S1–S5. A significant increase in the level of nine cytokines was observed in the ccRBCs compared to oRBCs. The significant changes between the oRBCs and the ccRBCs were observed for chemokines (IL-8, MCP-1 and GRO-α), growth factors (bFGF, M-CSF, SCF, and VEGF), pro-inflammatory cytokines (IL-18), and an anti-inflammatory cytokine (IL-13) (Fig. 1). The largest observed increase was for bFGF, where, ccRBCs contained 983 ± 414 pg/mL compared with 22 ± 18 pg/mL in oRBCs. Similarly, the concentration of GRO-α was 259 ± 214 pg/mL in the ccRBCs compared with 6 ± 6 pg/mL in oRBCs. These results indicate that the ccRBCs sequestered a variety of cytokines as a direct result of the presence of a cancer cell line in co-culture. The concentration of 4 of these cytokines (GRO-α, IL-8, MCP-1, and SCF) was detected in much higher levels in the conditioned media of A549 cells compared to what was quantified in the ccRBCs (Fig. 1). This may indicate a low binding capacity for these particular cytokines on RBCs, however, of these all but SCF are known ligands of DARC on RBCs^15^. Shen *et al*.^11^ similarly demonstrated that RBCs bound chemokines from the conditioned media of prostate cancer cells and they found that this removal of the chemokines by the RBCs was crucial in minimising tumour growth and tumour vessel density in an animal model. We hypothesised that as RBCs move through the tumour capillary network *in vivo*, they would accumulate tumour-associated cytokines and subsequently have downstream effects on the immune cells they come in contact with in the peripheral circulation. To investigate this, the effect of ccRBCs on T-cells was determined.

### Augmentation of Jurkat cell proliferation with ccRBCs

**Figure 2:**
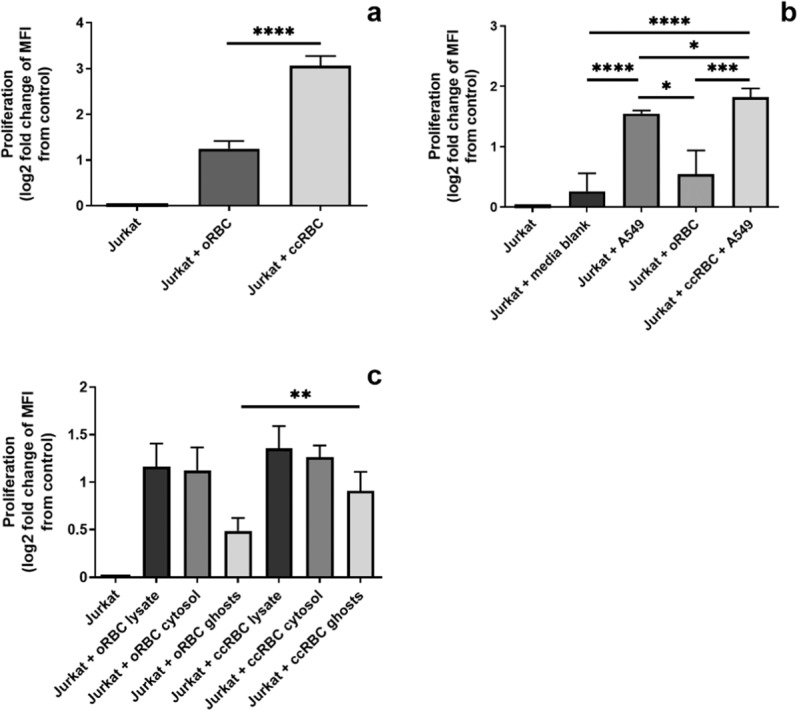
Jurkat proliferation following RBC treatment. Graphical representation of Jurkat cell proliferation after 6 days following treatment with (**a**) intact oRBCs or ccRBCs; (**b**) conditioned media from A549, oRBCs, or ccRBC + A549; or (**c**) lysate, cytosol, or membranes isolated from oRBCs and ccRBCs. Data are presented as fold change of loss of CFSE MFI from untreated Jurkat cells after 6 days in culture at 37 °C with 5% CO_2_ (mean ± SD, *n* = 5). Data are statistically significantly different if *p < 0.05, **p < 0.01, or ****p < 0.0001.

RBCs are known to promote T-cell proliferation *in vitro*^4,6^. We therefore aimed to assess the effect of ccRBCs on this rate of proliferation using the leukemic T-cell line, Jurkat cells. Proliferation of Jurkat cells was measured by staining with CFSE, which is a fluorescent dye that is incorporated into cells and the dye intensity (MFI) is reduced with each successive cell division. For intact RBCs, ccRBCs had the greatest effect on Jurkat proliferation with a mean decrease in MFI of 8.3-fold, whilst oRBCs only had a mean decrease in MFI of 2.4-fold compared to the control (Fig. 2a), indicating that ccRBCs stimulated the most cellular proliferation.

To assess if this increase in proliferation was mediated by soluble factors, the ccRBC + A549, oRBC, and A549 conditioned media was used to stimulate the proliferation of the Jurkat cells. No difference in proliferation was observed for Jurkat cells treated with the culture media blank or with oRBC conditioned media, that is, RBCs incubated alone (Fig. 2b). However, A549, and ccRBC + A549 conditioned media stimulated significantly more proliferation than the untreated Jurkat cells (*p* < 0.0001) with mean fold changes of 2.9 and 3.5 respectively (Fig. 2b). Of note, the ccRBCs + A549 conditioned media stimulated the Jurkat cells to proliferate significantly more (3.5-fold) than the conditioned media of either component alone (2.9-fold and 1.5-fold for A549 and oRBC conditioned media respectively) (Fig. 2b) thus suggesting that soluble mediators were involved in the proliferative response to ccRBCs.

The conditioned media from A549 and ccRBC induced some, but not all, the proliferation detected in the Jurkat cells in response to intact ccRBCs. To explore that effect in more detail ccRBCs and oRBCs were separated into lysates, cytosols, and membrane fractions and the effect on proliferation determined. Both ccRBCs and oRBCs stimulated Jurkat proliferation compared to the control (Fig. 2c), but with no significant difference observed between the ccRBC and oRBC groups for the lysate and cytosol samples. In contrast, membranes isolated from ccRBCs stimulated a 1.9-fold increase in proliferation in Jurkat cells compared to a 1.4-fold increase in proliferation induced by oRBCs (*p* = 0.003) (Fig. 2c). These results demonstrate that priming RBCs with cancer cells resulted in some modification of the cells and specifically the RBC membrane that made the membranes more immunomodulatory, however the largest proliferative response in Jurkat cells was detected in the presence of intact ccRBCs. In order to investigate if intact ccRBCs could also stimulate proliferation in an autologous model, T-cells from a PBMC population were collected and analysed.

### Promotion of CD8+ T-cell proliferation and protection against apoptosis

**Figure 3:**
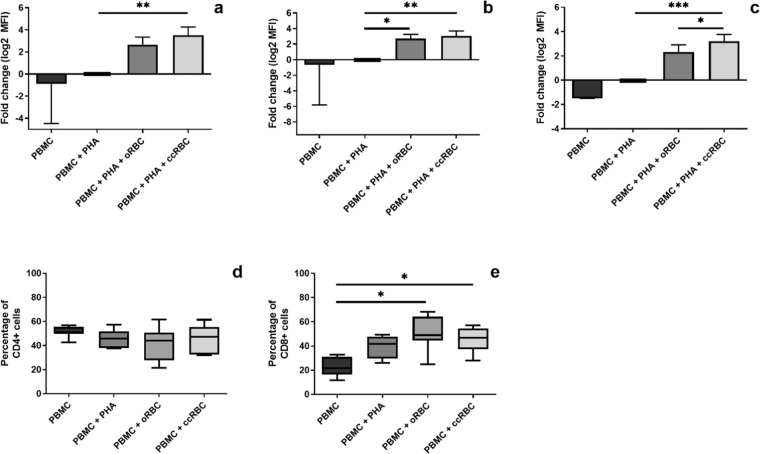
PBMC proliferation with RBC treatment. Figures represent proliferation of (**a**) CD3 + cells, (**b**) CD4 + cells, and (**c**) CD8 + cells from a PBMC population as fold change of proliferation from loss of CFSE fluorescence (MFI), and percentage of (**d**) CD4 + cells or (**e**) CD8 + cells in a CD3 + population of PBMCs following treatment with nothing (PBMC), with PHA-P (PBMC + PHA), with PHA-P and oRBCs (PBMC + PHA + oRBC), or with PHA-P and ccRBCs (PBMC + PHA + ccRBC). Proliferation index presented as mean ± SD and percentages presented as box and whisker plots with median (*n* ≥ 6). PBMCs were analysed by flow cytometry after 5 days in culture at 37 °C with 5% CO_2_. Data are statistically significantly different if *p < 0.05, **p < 0.01, or ***p < 0.001.

Intact ccRBCs stimulated the highest level of proliferation in the Jurkat cells, thus it was of interest to see if these results could be replicated in an autologous mixed cell model. This was achieved by monitoring the proliferation of T-cells (CD3+) and subsets of T-cells (CD4+ and CD8+ cells) using CFSE staining in the presence of intact ccRBCs or oRBCs. A small number of papers have investigated the effect of RBC treatment on the proliferation of cells in a PBMC population. In these papers, a consistent increase in proliferation of PBMCs following treatment with naïve RBCs has been reported^2,4–6,16,17^. In this study, treatment with autologous ccRBCs or oRBCs stimulated increased proliferation compared to PBMCs treated with PHA-P alone. This result was consistent for CD3+, CD4+, and CD8+ cells (Fig. 3a–c). Moreover, the ccRBCs promoted the proliferation of CD8+ T-cells significantly more than the oRBCs (Fig. 3c). Examples of the flow cytometry histograms and dot plots for this analysis can be found in Supplementary Figs. S6–S8. In support of this, an analysis of cell numbers within a CD3+ population revealed that the number of CD4+ cells, unlike CD8+ cells, did not change with PHA-P stimulation or with ccRBC or oRBC treatment, whilst the number of CD8+ cells did increase with both ccRBC and oRBC treatment (Fig. 3d,e). This increase in the number of cytotoxic T-cells (CD8+ cells) following RBC treatment has been reported previously in both PBMC cultures and purified CD3+ cultures^3,4^. It is not clear if the reason for this selective expansion of CD8+ cells *in vitro* is simply a result of a faster doubling time than CD4+ cells or if it was true preferential expansion.

**Figure 4:**
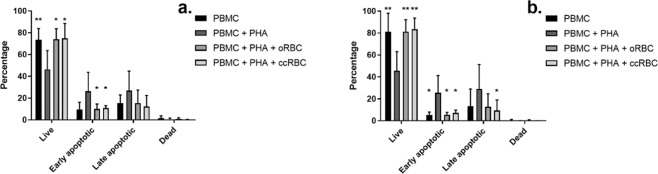
PBMC apoptosis following RBC treatment. Percentage of live (Annexin V−/PI−), early apoptotic (Annexin V + /PI−), late apoptotic (Annexin V+/PI+), and dead (Annexin V−/PI+) cells in (**a**) CD4+ populations or (**b**) CD8+ populations. PBMCs were treated with nothing (PBMC), with PHA-P (PBMC + PHA), with PHA-P and oRBCs (PBMC + PHA + oRBC), or with PHA-P and ccRBCs (PBMC + PHA + ccRBC) for 5 days at 37 °C and 5% CO_2_ (mean ± SD, *n* = 5). Data are statistically significantly different from PBMCs treated with PHA-P if *p < 0.05, or **p < 0.01.

PHA-P stimulates PBMC proliferation but also induces apoptosis in those same cells. Apoptosis of PBMCs was measured by Annexin V/PI co-staining and flow cytometry analysis. The percentage of live cells dropped significantly from a mean of 73% or 81% for CD4+ and CD8+ cells respectively in the untreated group, to 46% in the PHA-P stimulated group for both CD4+ and CD8+ cells (Fig. 4). However, cell survival was recovered in the presence of the ccRBCs or oRBCs, for both CD4+ and CD8+ cells (Fig. 4). This protective activity of RBCs on PBMCs has been shown to be cell-cell mediated, independent of monocyte activity and to specifically affect T-cells^4,6,16,17^. Unlike oRBCs, the inclusion of ccRBCs resulted in significantly fewer late apoptotic CD8+ cells (Annexin V+/PI+) compared to the PHA-P stimulated group (Fig. 4b). However, there is little difference in the means of these groups (oRBC: 12.7%; ccRBC: 9.3%).

### RBCs modulate T-cell activation

**Figure 5:**
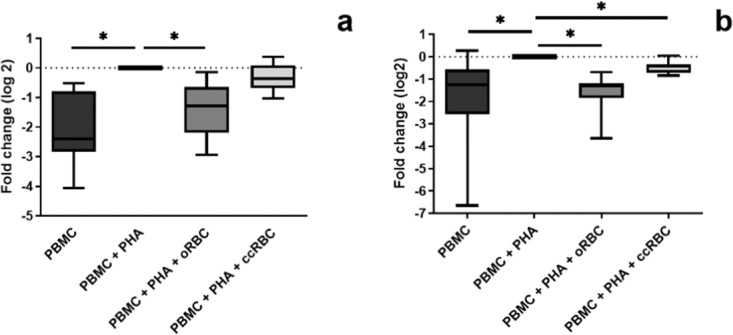
Expression of transcription factors in T-cells following RBC treatment. Expression of transcription factors in CD8+ population of PBMCs following treatment with nothing (PBMC), with PHA-P (PBMC + PHA), with PHA-P and oRBCs (PBMC + PHA + oRBC), or with PHA-P and ccRBCs (PBMC + PHA + ccRBC) after 5 days in culture at 37 °C and 5% CO_2_ (median ± min/max, *n* = 7). Data are presented as fold change from PBMCs treated with PHA-P in expression of (**a**) GATA-3 and (**b**) T-bet. Data are statistically significantly different from PBMCs + PHA if *p < 0.05.

With the observation that the ccRBCs stimulated proliferation of CD8+ cells, it was hypothesised that these cells were being driven down a particular differentiation pathway. Thus, to elucidate if there was any shift towards either a T_C_1 (immuno-potentiating) or T_C_2 (immunosuppressive) profile following RBC treatment, the expression of two transcription factors and the cytokine secretion profile of PBMCs was assessed. Upregulation of T-bet is typically indicative of a T_C_1 profile, and upregulation of GATA-3 is indicative of a T_C_2 profile in T-cells^18,19^. Contrary to the hypothesis, the results of this study did not illustrate differentiation of CD8+ cells into one subtype or another, but instead showed the overall activation of these cells. Stimulation with PHA-P alone promoted the expression of both GATA-3 and T-bet in CD8+ T-cells in comparison to the unstimulated control (Fig. 5). Treatment with oRBCs in conjunction with PHA-P supressed the expression of both transcription factors such that it resembled the expression of unstimulated PBMCs. In contrast, ccRBC treatment instead resulted in a very similar profile of activation observed with PHA-P alone (Fig. 5). When compared to the PHA-P sample, the mean fold change in GATA-3 expression was a reduction of 2.1-fold for oRBC treatment and only 1.2 for ccRBC treatment. Similarly, the mean fold change in T-bet expression was a reduction of 2.7-fold for oRBC treatment and only 1.4 for ccRBC treatment.

**Figure 6:**
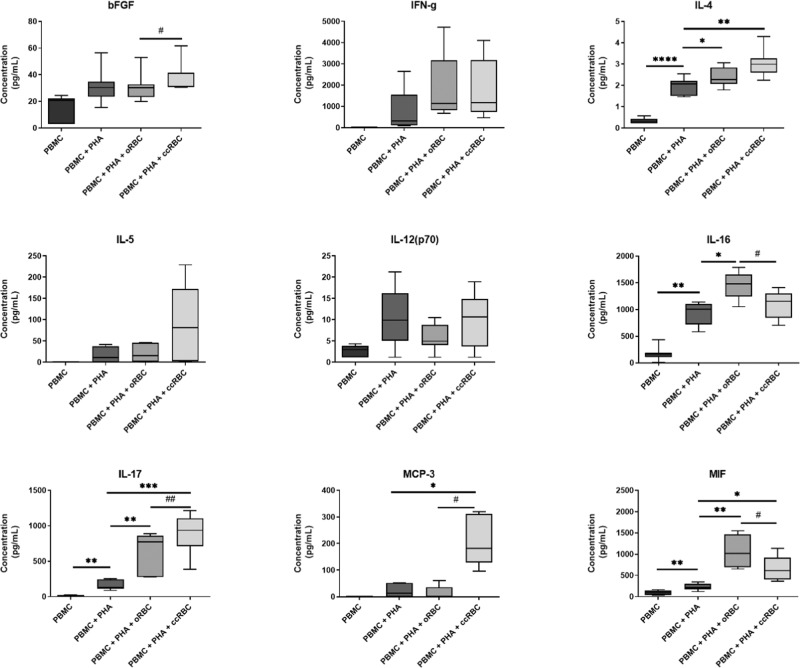
Secretion of cytokines from PBMCs following RBC treatment. Summary of cytokines in PBMC conditioned media from untreated PBMCs (PBMC), PBMCs treated with PHA-P (PBMC + PHA), PBMCs treated with PHA-P and oRBCs (PBMC + PHA + oRBC), and PBMCs treated with PHA-P and ccRBCs (PBMC + PHA + ccRBC) as measured by Bio-Plex. Data are presented as box and whisker plots with median concentration (n = 5). Data are statistically significantly different from PBMCs treated with PHA-P if *p < 0.05, **p < 0.01, ***p < 0.001, or ****p < 0.0001 and PBMC + PHA + oRBCs are statistically significantly different from PBMC + PHA + ccRBCs if ^#^p < 0.05.

In addition to the expression of transcription factors, cytokine release from CD8+ T-cells can be indicative of a shift towards a specific differentiation subtype. As such, the cytokines in the PBMC conditioned media were quantified using Bio-Plex analysis. The fold change of a representative selection of cytokines is presented in Fig. 6; the data for the remaining cytokines can be found in Supplementary Figs. S9–S13. Treatment with PHA-P alone significantly stimulated increased secretion of 30 cytokines. Of these, 18 were significantly increased with the addition of oRBCs and 16 significantly increased with ccRBCs (Supplementary Figs. S9–S13). ccRBCs altered the secretion profile in comparison to the oRBCs with significant differences observed for five cytokines; IL-17 and MIF, pro-inflammatory cytokines, IL-16 and MCP-3, chemokines, and bFGF, a growth factor (Fig. 6). Secretion of INF-γ or IL-12 is correlated to a T_C_1 subtype, and secretion of IL-4 and IL-5 is correlated to a T_C_2 subtype of CD8 + T-cells. In this study, the secretion of T_C_1 cytokines was low and the levels did not change significantly between the oRBC and ccRBC groups (Fig. 6). T_C_2 cytokines on the other hand, trended toward an increased secretion change with ccRBCs (Fig. 6). IL-4 and IL-5 were released with a trend to higher concentrations from the PBMCs treated with ccRBCs, than those treated with oRBCs (Fig. 6).

### Level of haemolysis in conditioned media

**Figure 7:**
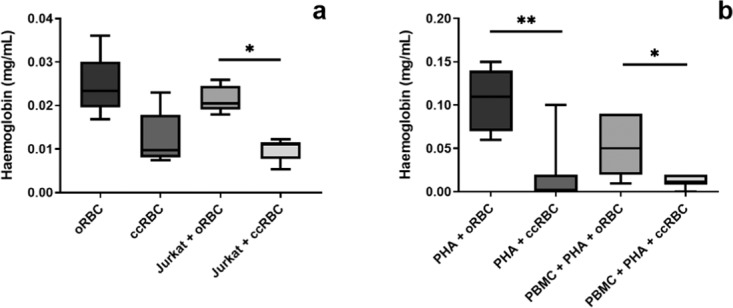
RBC haemolysis in conditioned media. Concentration of haemoglobin in the conditioned media of oRBCs or ccRBCs (2.5 × 10^6^ RBCs per condition) incubated (**a**) alone or with Jurkat cells for 6 days at 37 °C with 5% CO_2_ or (**b**) with PHA or with PBMC + PHA for 5 days at 37 °C with 5% CO_2_. Haemoglobin concentration was measured by absorbance at 414 nm. Data are presented as box and whisker plots with median, *n* ≥ 5, statistically significantly different *p < 0.05, or **p < 0.01.

The increased concentration of cytokines in the PBMC conditioned media contained ccRBCs and oRBCs could not be wholly attributed to ccRBC or oRBC release or lysis, but is instead active secretion from the PBMCs. As an example, PBMCs treated with PHA-P alone released 153 ± 67 pg/mL of IL-17 into the conditioned media which was significantly lower than the amount released from PBMCs treated with oRBCs or ccRBCs aswell (Fig. 6). oRBCs incubated alone under the same conditions released 7.5 ± 1.1 pg/mL of IL-17 whilst PBMCs treated with oRBCs released 645 ± 268 pg/mL. Similarly, the ccRBC equivalent released 7.0 ± 5.1 pg/mL when incubated alone, or 891 ± 282 pg/mL when PBMCs were treated with the ccRBCs. Thus, the concentration of IL-17 released by RBCs alone does not explain the observed increase. To investigate the extend of haemolysis that occurred over the culture period, the concentration of haemoglobin in the Jurkat and PBMC conditioned media was quantified by monitoring the absorbance of the solutions at 414 nm. These results revealed that the presence of Jurkat cells or PBMCs had no effect on the level of the detectable haemolysis (Fig. 7) and the concentration of haemoglobin in the conditioned media samples corresponded to the lysis of between 3 and 15% of the total RBC number for the Jurkat cell conditioned media, and between 1 and 9% for the PBMC cell conditioned media. Of note, the level of haemolysis was significantly lower in the ccRBC group in comparison to the oRBC group for both the Jurkat and PBMC conditioned media (Fig. 7) which may be a result of Gardos channel activity on the ccRBCs which can be activated in the presence of cytokines.

## Discussion

The role that RBCs play in modulating the activity of immune cells is now beginning to be reported^1,4,17^ and a small number of papers have outlined how RBCs can become dysregulated in inflammatory conditions or following long term storage^7,20,21^. Our study aimed to build on these initial reports of RBCs derived from either healthy or disease cohorts by intentionally manipulating RBCs through co-culture with cancer cells. This study reproduced the findings of the prior reports, wherein naïve RBCs promoted the proliferation of T-cells (Figs. 2 and 3) and protected them from apoptosis (Fig. 4). This, however, is the first study to outline (1) the cytokine changes that can occur to RBCs with co-culture modification, and (2) the subsequent altered or amplified response of the T-cells to the ccRBCs as compared to the unmanipulated RBCs (oRBCs). A broad goal of this research was to model the communication between proliferating tumour cells and RBCs *in vivo* and to observe any resulting downstream effects those RBCs would have on T-cells.

RBCs have been described as potential sinks for inflammatory cytokines^8,22,23^. In support of this hypothesis, this study found that RBCs were capable of binding cytokines released from an adherent tumour cell line (Fig. 1). In the RBCs co-cultured with the NSCLC cell line - A549 cells - (ccRBCs) the levels of nine cytokines were significantly higher in these ccRBCs including IL-8, bFGF, and VEGF (Fig. 1). These particular cytokines have been implicated as important in the progression of NSCLC. Zhao *et al*. reported that NSCLC cells express various cytokines that promote tumour growth and angiogenesis, including VEGF and bFGF^24^ and both of these cytokines have been correlated with poor patient prognosis^25^. Intracellular bFGF promotes NSCLC tumour growth and stimulates secretion of VEGF^26^. The ccRBCs also contained significantly higher levels of three chemokines, IL-8, GRO-α and MCP-1 (Fig. 1). Similarly, these have also been implicated in tumour growth and angiogenesis^27–29^. These results demonstrate that following cell-to-cell contact, RBCs could be loaded with a group of pro-tumorigenic proteins. Shen *et al*. investigated the role of DARC on RBCs on cytokine binding in a model of prostate cancer^11^. Similar to our study, they identified increased binding of IL-8 and GRO-α in the presence of RBCs, but unlike this study, the concentration of VEGF was unchanged. Although in each study the RBCs were being modified by a secondary cell line, the final cytokine profile was slightly different. Thus, the resulting cytokine profile of these RBCs is likely to be dependent upon which secondary cell line is used. Analysis of the cytokine profile of RBCs isolated from patients may be a useful source for the identification of disease specific biomarkers.

This ability of RBCs to bind cytokines, may also be a clue to explaining why ccRBCs were less susceptible to haemolysis than the oRBCs (Fig. 7). The Gardos channel is a potassium channel on RBCs that is responsible for maintaining cell volume, and has more recently been shown to be regulated by cytokine binding to DARC^30^. A number of the cytokines that bound in high concentrations to the ccRBCs are also responsible for regulating this channel (including IL-8 and RANTES)^30^. High levels of these cytokines or high expression of DARC is correlated with increased dehydration in RBCs^31^ which may be the mechanism behind the lower level of haemolysis observed in the ccRBCs. Gardos channel activation in RBCs has been shown to be protective against haemolysis^32^.

The effect of the ccRBCs on the proliferation and survival of T-cells was of key interest in this study. T-cell proliferation is typically used as a model of immune cell activation. Upon presentation with an antigen or a stimulatory signal, T-cells undergo clonal expansion and cell differentiation^33^. Apoptosis of these cells is a crucial process for maintaining homeostasis following immune stimulation^34^. In this study it was demonstrated that inclusion of intact oRBCs in culture with a leukemic T-cell line (Jurkat cells) or autologous PBMCs stimulated proliferation, and the addition of ccRBCs stimulated significantly more expansion (Figs. 2a and 3c). Notably, both oRBCs and ccRBCs were found to be protective of activation-induced apoptosis (Fig. 4) and there were no significant differences between these two conditions. Previous reports have identified a correlation between RBC-induced proliferation and RBC-induced protection against apoptosis^2^. Since both oRBCs and ccRBCs stimulated T-cell proliferation, although by different amounts, it is likely that this is the reason that both conditions were found to be protective against apoptosis. Previous studies have reported that the immunogenic activity of naïve RBCs was mediated by soluble protein factors and that these factors were present in the non-vesicle fraction of the RBC conditioned media^16^. The increase in proliferation of Jurkat cells that was observed with the conditioned media in this study was likely also a result of a change in one or more soluble factors and this change was dependent upon the inclusion of RBCs in the initial culture (Fig. 2b). The membranes of the ccRBCs were also more immunostimulatory than the membranes from the oRBCs (Fig. 2c). As some inflammatory cytokines are known to bind to specific receptors on the RBC membrane^8,35^, these results together support the observation that one or more soluble factors, such as cytokines, may be mediating the stimulatory activity of RBCs.

It should be noted, that the PBMC analysis in this study was performed on PBMCs stimulated with PHA-P. This particular stimulant was selected as it had been previously identified as being able to produce the largest proliferative effect when used in conjunction with RBCs compared with other stimulants^17^. However, as observed in this study PHA-P can produce a negative effect on T-cells, notably promoting apoptosis (Fig. 4), as well as its ability to promote T-cell proliferation. It is also a broad target stimulant with RBC agglutination properties. For these reasons, its use was a limitation in this current study, but one we accepted as it would equally affect both oRBC and ccRBC treatment which was our primary comparison of interest. Future studies however, may benefit from using more applicable T-cell stimulants such as anti-CD3/CD28 which are less deleterious to the cells.

Nevertheless, the promotion of Jurkat proliferation in the presence of ccRBCs was modelled with autologous PBMCs, and a particular increase in the number of CD8+ T-cells was observed (Fig. 3c). Interestingly, in patients receiving multiple RBC transfusions, a similar preferential expansion of CD8+ T-cells has been reported^36,37^. In one study, participants receiving multiple transfusions for sickle cell anaemia or haemophilia had significantly higher levels of CD8+ T-cells than non-transfused controls^36^. It has been suggested that this preferential CD8+ cell proliferation may be one of the reasons for the immunosuppression that is observed following RBC transfusions^38^. This transfusion-induced immunosuppression appears to be beneficial for specific indications, for example, it has been reported that administration of RBC transfusions prior to renal transplant surgery was strongly correlated with improved graft survival^39^. In cancer, however, the administration of RBC transfusions has been reported to promote immunosuppression and subsequently disease progression^40^. The selective proliferation of CD8+ T-cells following ccRBC treatment in this study may too be demonstrative of the development of this immunosuppressive environment. The cytokines detected in the conditioned media of the PBMCs treated with ccRBCs also indicated the development of an immunosuppressive profile, with increased secretion of T_C_2 cytokines and no change in the T_C_1 cytokines in comparison to the oRBC group (Fig. 6). This shift towards an immunosuppressive environment, however, was not detected in the expression of T-bet and GATA-3. Instead, the presence of either ccRBCs or oRBCs in culture with the PBMCs had an effect on the overall activation of the CD8+ T-cells. That is, the expression of both of these transcription factors was attenuated following treatment with oRBCs, thus suggesting a role for RBCs in regulating immune activation and differentiation. This role, however, was dysregulated with ccRBCs for both T-bet and GATA-3 (Fig. 5). This is the first report on the expression of GATA-3 and T-bet in a CD8+ T-cell population following treatment with RBCs. Buttari *et al*. demonstrated that RBCs from carotid atherosclerosis patients were also dysregulated and were no longer able to promote maturation of dendritic cells^20^. Our results provide further evidence that RBCs can be functionally altered in their ability to interact with immune cells.

These results also demonstrated that RBCs isolated from healthy individuals could be manipulated to alter their cytokine profile and their influence on T-cells. Similar dysregulation of RBC activity has been demonstrated in disease (carotid atherosclerosis)^6^, following surgery^7^, or following haemodialysis^41^. Further investigation around what causes this dysregulation *in vivo* and what role it may play in disease will be valuable. The experiments in this study were not designed to perfectly model the role of RBCs in NCSLC. However, they do suggest that if there is interaction between the RBCs and cancer cells there may be some downstream immunological effects *in vivo*. In order to validate this, we plan to isolate RBCs from NSCLC participants to elucidate if their interaction with immune cells are different to healthy controls. NSCLC currently has a poor prognosis and a low median survival time^42^. Thus, further understanding of the biology of the condition may be valuable in identifying novel therapeutic targets or improving early detection in at-risk groups. RBCs isolated from healthy individuals can modulate immune cells by promoting T-cell proliferation, protecting these same cells from apoptosis, and suppressing stimulant-driven activation of CD8+ T-cells. These results support the hypothesis that RBCs play a key role in modulating the immune system. In addition, this study highlights that RBCs and their interactions with the immune system are malleable. A dysregulation in RBC activity has been suggested in disease and has now been observed through intentional alteration of the cells and their cytokine profile by exposing them to a NSCLC cell line. In the presence of these altered RBCs, T-cells were stimulated to proliferate to a greater degree, were no longer protected from stimulant-driven activation, and were driven to release a variety of cancer-related cytokines. This study identifies the potential for RBC involvement in disease or in their use as disease biomarkers.

## Materials and methods

All methods were carried out in accordance with relevant guidelines and regulations. All experimental protocols were approved by the Northern Sydney Coast Human Research Ethics Committee of NSLHD and CCLHD (reference: 1201-046 M). Informed consent was obtained from all subjects.

### Reagents and mAbs

High molecular weight dextran (450–650 kDa), DMEM, RPMI-1640, bovine serum albumin (BSA), RBC lysis buffer, 5(6)-carboxyfluorescein diacetate *N*-succinimidyl ester (CFSE), phytohemagglutinin-P, from *Phaseolus vulgaris* (PHA-P), and human derived haemoglobin were acquired from Sigma-Aldrich (St. Louis, MO). Antibiotic-antimycotic (ABAM), penicillin-streptomycin (10,000 U/mL), L-glutamine (200 mM solution), and propidium iodide (PI) were from Life Technologies (Carlsbad, CA). Ficoll-Paque was from GE Healthcare (Little Chalfont, United Kingdom), and fetal bovine serum was from AusGeneX (Gold Coast, Australia). Anti-human CD3-APC, CD3-FITC, CD4-APC, CD8a-APC, GATA 3-PE, T bet-PE, corresponding anti-mouse isotype controls (Table 1), Annexin V-FITC labelling kit, and Nuclear Factor Fixation and Permeabilisation Buffer Set were from BioLegend (San Diago, CA). BD Calibrite beads were from BD Biosciences (San Jose, CA).

**Table 1:** Antibodies and proteins used for immunofluorescence staining of PBMCs.

Antibody/Protein	Fluorochrome	Clone
Anti-human CD3 (Mo IgG1, κ)	APC	SK7
Anti-human CD3 (Mo IgG1, κ)	FITC	UCHT1
Anti-human CD4 (Mo IgG1, κ)	APC	RPA-T4
Anti-human CD8a (Mo IgG1, κ)	APC	HIT8a
Anti-human GATA-3 (Mo IgG2b, κ)	PE	16E10A23
Anti-human T-bet (Mo IgG1, κ)	PE	4B10
Annexin V	FITC	—
Isotype control (Mo IgG1, κ)	FITC	MOPC-21
Isotype control (Mo IgG1, κ)	APC	MOPC-21
Isotype control (Mo IgG1, κ)	PE	MOPC-21
Isotype control (Mo IgG2b, κ)	PE	MPC-11

### Blood donation

Whole blood was collected in two or more K_2_EDTA vacutainers (BD Biosciences) from healthy volunteers by venepuncture (*n* = 12; female: 7, male: 5) and were subsequently used to isolated autologous RBCs and PBMCs. Blood was collected and processed at room temperature within one hour of collection. This study was approved by the Northern Sydney Coast Human Research Ethics Committee of NSLHD and CCLHD (1201–046 M). Written consent was collected from all participants before participation in this study.

### RBC isolation

One vacutainer of whole blood was centrifuged (1500 *g*, 10 minutes) and the upper plasma and buffy coat layer discarded. The remaining cells was resuspended in an equal volume of sodium chloride (0.15 M). Dextran (6% w/v in 0.15 M sodium chloride) was then added at a 1:4 ratio (dextran:cell suspension) and the suspension was allowed to sediment at room temperature for 30 minutes. The lower RBC fraction was isolated, washed once in phosphate buffered saline (PBS), and cell numbers and purity of RBCs were determined using a haematology analyser (Coulter Act Diff, Beckman Coulter, Brea, CA). The remaining RBCs were used for incubation experiments or were frozen at −80 °C in PBS. Frozen RBCs were subjected to three freeze-thaw cycles to ensure complete cellular lysis and lysates were clarified by centrifugation to remove particulates (2000 *g*, 5 minutes).

### Peripheral mononucleated cell (PBMC) isolation and culture

Autologous PBMCs were isolated from whole blood using standard Ficoll-Paque density gradient separation. Briefly, anticoagulated blood was diluted in an equal volume of PBS and was layered on top of Ficoll-Paque (2:1 ratio respectively) and centrifuged without a brake (400 *g*, 40 minutes). After centrifugation, the buffy coat and was washed once with PBS (400 *g*, 5 minutes). Any contaminating RBCs were lysed with RBC lysis solution (Sigma-Aldrich) for 10 minutes at 37 °C. PBMCs were then put into culture in RPMI-1640 with 10% FBS, 100 U/mL penicillin-streptomycin, and 2 mM L-glutamine at 37 °C, 5% CO_2_. After three days of culture, cells in suspension were collected for use in proliferation assays, thus depleting adherent monocytes.

### RBC priming with A549 cells

A549 cells were seeded at 0.1 × 10^6^ cells per mL of culture media (DMEM, 10% FBS, 1% ABAM) and left to adhere to the flask for 24 hours (37 °C, 5% CO_2_) after which the culture media was replaced. To produce modified RBCs (ccRBCs), RBCs were added to these adherent cells at 100:1 respectively (10 × 10^6^ RBCs/mL) and were incubated for 72 hours (37 °C, 5% CO_2_). oRBCs were produced by incubating RBCs for 72 hours in the same culture media without A549 cells (10 × 10^6^ RBCs/mL, 37 °C, 5% CO_2_), and A549 cells were incubated alone under the same conditions to produce A549 conditioned media. Following incubation, the ccRBC + A549, oRBC, and A549 conditioned media was collected and the ccRBCs and oRBCs were collected by centrifugation out of the conditioned media (1000 *g*, 10 minutes). Any remaining particulates in the conditioned media were removed by centrifugation (2000 *g*, 10 minutes) after which it was stored at −80 °C. The ccRBCs and oRBCs were washed once with PBS and were collected for use in further experiments or were stored at −80 °C to produce lysates. In addition to the lysates, ccRBC and oRBC cytosol and membranes were isolated by centrifugation of the lysates (16,000 *g*, 20 minutes). After centrifugation the cytosol was collected and the resulting membranes were washed twice with Milli-Q water (16,000 *g*, 20 minutes) before being resuspended in RPMI-1640 media.

### Jurkat cell culture

Jurkat cells were cultured in RPMI-1640 with FBS (10% or 2.5%), 100 U/mL penicillin-streptomycin, and 2 mM L-glutamine. Jurkat cells were seeded at 1 × 10^5^ cells/mL and grown to 1 × 10^6^ cells/mL with 10% FBS then were sub-cultured into 2.5% FBS and dead cells were removed after five days by resuspending the cells in PBS, layering the cell suspension over Ficoll-Paque, and centrifuging to isolate the live cells at the interface of Ficoll-Paque and PBS (400 *g*, 40 minutes, no bake). The proliferation assay was initiated after a total of six days in culture with 2.5% FBS.

### Proliferation assay

Jurkat cells or PBMCs (1 × 10^6^ cells/mL) were stained with CFSE (5 µM in PBS) for 10 minutes at 37 °C. After staining, cells were washed once with FBS to quench excess CFSE. Immediately after labelling, cells were analysed by flow cytometry and staining efficiency was determined to be greater than 99%. Proliferation assays were performed using CFSE stained Jurkat cells or PBMCs for up to 6 days in low serum media (RPMI-1640, 1% FBS, 100 U/mL penicillin-streptomycin, 2 mM L-glutamine). Jurkat cells (0.1 × 10^6^ cells/mL) were cultured for 6 days and PBMCs (0.3 × 10^6^ cells/mL) were cultured for 5 days with autologous ccRBCs or oRBCs at a ratio of 1:10 respectively (37 °C, 5% CO_2_). PBMCs were also stimulated with PHA-P (5 µg/mL). In variations of the proliferation assay, Jurkat cells were treated with RBC lysates, cytosols, or membranes from ccRBCs or oRBCs at concentrations equivalent to 0.1 × 10^6^ cells/mL or were treated with A549, oRBC, or ccRBC + A549 conditioned media (undiluted). For all samples, the PBMC conditioned media was collected and stored at −80 °C for cytokine analysis. The PBMCs and Jurkat cells were isolated and processed for flow cytometry analysis as outlined below.

### Cell staining and flow cytometry

For each sample, 1 × 10^4^ events of the stained target population were acquired and analysed by flow cytometry using Cell Quest Pro software (ver. 5.2.1). For cells treated with ccRBCs or oRBCs, the remaining RBCs were lysed with RBC lysis buffer (Sigma-Aldrich, 10 minutes, 37 °C). Following culture, Jurkat cells were isolated by centrifugation (1000 *g*, 10 minutes). The Jurkat cells were then washed in PBS before being analysed by flow cytometry (FACScalibur) to monitor level of CFSE fluorescence. Following culture, the PBMCs were isolated by centrifugation (1500 *g*, 10 minutes). The PBMCs were then washed once in PBS before commencing staining with the relevant monoclonal antibodies. Cells stained with CFSE were only co-stained with APC conjugated antibodies, unstained PBMCs were used for all other antibodies. PBMCs were stained in two steps, with extracellular markers in the first step (CD3, CD4, CD8, or corresponding isotype controls) using staining buffer (PBS, 0.5% BSA) and intracellular markers in the second step (T-bet, and GATA-3, or corresponding isotype controls) using Nuclear Factor Fixation and Permeabilisation Buffer Set according to manufacturers instructions. Cell staining was performed at 4 °C in the dark. Multi-colour flow cytometry (FACScalibur) was used to monitor the level of CFSE fluorescence in specific cellular populations and to monitor the expression of transcription factors in CD8+ populations. For the purposes of compensation, BD Calibrite beads and single colour controls for CFSE, FITC, PE, and APC were used to optimise detection. The proliferation index of the CFSE labelled PBMCs was determined by monitoring the percentage of cells in each generation as previously described^43^. Following cell surface staining, PBMCs were labelled with Annexin V labelling kit with PI according to manufacturers instructions. After labelling, the cells were analysed immediately by flow cytometry.

### Protein detection

In this study, two multiplex assays were utilised. The first was the 27-plex human cytokine panel that assays for FGF basic, Eotaxin-1, G-CSF, GM-CSF, IFN-γ, IL-1β, IL-1ra, IL-2, IL-4, IL-5, IL-6, IL-7, IL-8, IL-9, IL-10, IL-12(p70), IL-13, IL-15, IL-17, IP-10, MCP-1, MIP-1α, MIP-1β, PDGF-BB, RANTES, TNF-α, and VEGF, and the second was the 21-plex human cytokine panel that assays for IL-1α, IL-2Ra, IL-3, IL-12(p40), IL-16, IL-18, CTACK, GRO-α, HGF, IFN-α2, LIF, MCP-3, M-CSF, MIF, MIG, β-NGF, SCF, SCGF-β, SDF-1α, TNF-β, TRAIL (Bio-Plex Pro 27-plex and 21-plex, Bio-Rad). The assays were performed according to manufacturer’s instructions using an automated magnetic wash station (Bio-Plex Pro II, Bio-Rad) for the washing steps. The assays were run on the Luminex 200 system (Bio-Rad) and fluorescence values were collected. The calibration curve for each cytokine was analysed with 5 parametric logistic curve regression using Bio-Plex manager software (ver. 5.0, Bio-Rad, USA). Standard values were considered acceptable if the points fell within 80–120% of the expected values. Levels of free haemoglobin in plasma samples and conditioned media samples were monitored by assessing absorbance at 414 nm (Synergy 2 Multi-Mode plate reader, BioTek, Winooski, VT) as previously described^44^. Peaks at this wavelength are indicative of free haemoglobin. RBC membranes were removed from the conditioned media samples prior to analysis by centrifugation at 16,000 *g* for 15 minutes. A haemoglobin calibration curve was prepared using human-derived haemoglobin at known concentrations and was analysed on GraphPad Prism software (ver. 6, USA).

### Statistical analysis

Comparison of multiple treatment groups were statistically evaluated using a one-way ANOVA with correction for multiple comparisons to assess statistical significance. Data were statistically significant if *p* < 0.05. Graphing of results was performed using GraphPad Prism software (ver. 6, USA) and Flowing Software (ver. 2.5.1) was used to create data histograms and dot plots for flow cytometry data and for data analysis. For cytokine analysis, statistical analysis of raw fluorescence responses was performed using ‘R’ version 3.2.3 (2015-12-10, R: A Language and Environment for Statistical Computing). Mixed-effects modelling was done using lmer^45^. The significance of interactions terms and interaction means and their associated standard errors were obtained using the Phia package^46^, for post-hoc analysis. Multiple test correction was done according to Holm’s method^47^. Further detail on the statistical analysis is included in the Supplementary methods.

## Supplementary information

Supplementary information.

## Data Availability

All data generated or analysed during this study are included in this published article (and its Supplementary Information files).
