# Supplementary material for: Red blood cells exposed to cancer cells in culture have altered cytokine profiles and immune function
Source: Sci Rep. 2020 May 7;10:7727. doi: 10.1038/s41598-020-64319-3 (PMC7206002; doi:10.1038/s41598-020-64319-3)
Supplement: Supplementary file 1 — Supplementary information. [file 41598_2020_64319_MOESM1_ESM.docx]

**Red blood cells exposed to cancer cells in culture have altered cytokine profiles and immune function.**

Authors: Elisabeth Karsten^1,2,3^; Edmond Breen^4^; Sharon A. McCracken^5^; Stephen Clarke^2,6^; Benjamin R. Herbert^1,3^

Affiliations: ^1^Translational Regenerative Medicine Laboratory, Kolling Institute, Royal North Shore Hospital, Sydney, Australia; ^2^Northern Clinical School, Faculty of Medicine, The University of Sydney, Sydney, Australia; ^3^Sangui Bio Pty Ltd, Sydney, Australia; ^4^Bioinformatic Consulting, Sydney, Australia; ^5^Perinatal Research, Kolling Institute, Royal North Shore Hospital, Sydney, Australia; ^6^Cancer Services, Northern Sydney Local Health District, St. Leonards, Sydney, Australia

Correspondence: Elisabeth Karsten, Sangui Bio Pty Ltd, PO Box 4054, Royal North Shore Hospital, Sydney, NSW 2065, Australia; e-mail: elisabeth@sanguibio.com

**Supplementary methods**

**Statistical analysis**

The cytokine data was analysed according to the following mixed-effects model, in R notation, was used: log2(FI)~Cytokine*Treatment + (1|kit:ID). The analysis was performed separately for RBCs lysates and conditioned media samples. Where the log2 of fluorescence responses (Fl) was modelled using 2-way mixed effects ANOVA, (1) Cytokine (48 levels), and (2) Treatment (2 levels for RBC lysates: oRBCs, or ccRBCs; 4 levels for PBMC conditioned media: none, PHA, oRBCs, or ccRBC) plus their interactions, together with one random term defined as (1|kit:ID), where ID represented subject identifier and where kit represented the 27-plex and 21-plex cytokine panels (2 levels). The random effects account for patient-to-patient variability and for differences with respect to Treatment and Fluid groupings across the 2 cytokine plates. The random effects account for patient-to-patient variability and for differences with respect to kit groupings across the 2 cytokine plates; it also accounted for the non-independence in the data due to multiple samples per subject. For the purposes of this manuscript, the term ‘significant’ will be used to indicate a statistically significant result of *p* < 0.05 unless otherwise specified.

**Supplementary results**


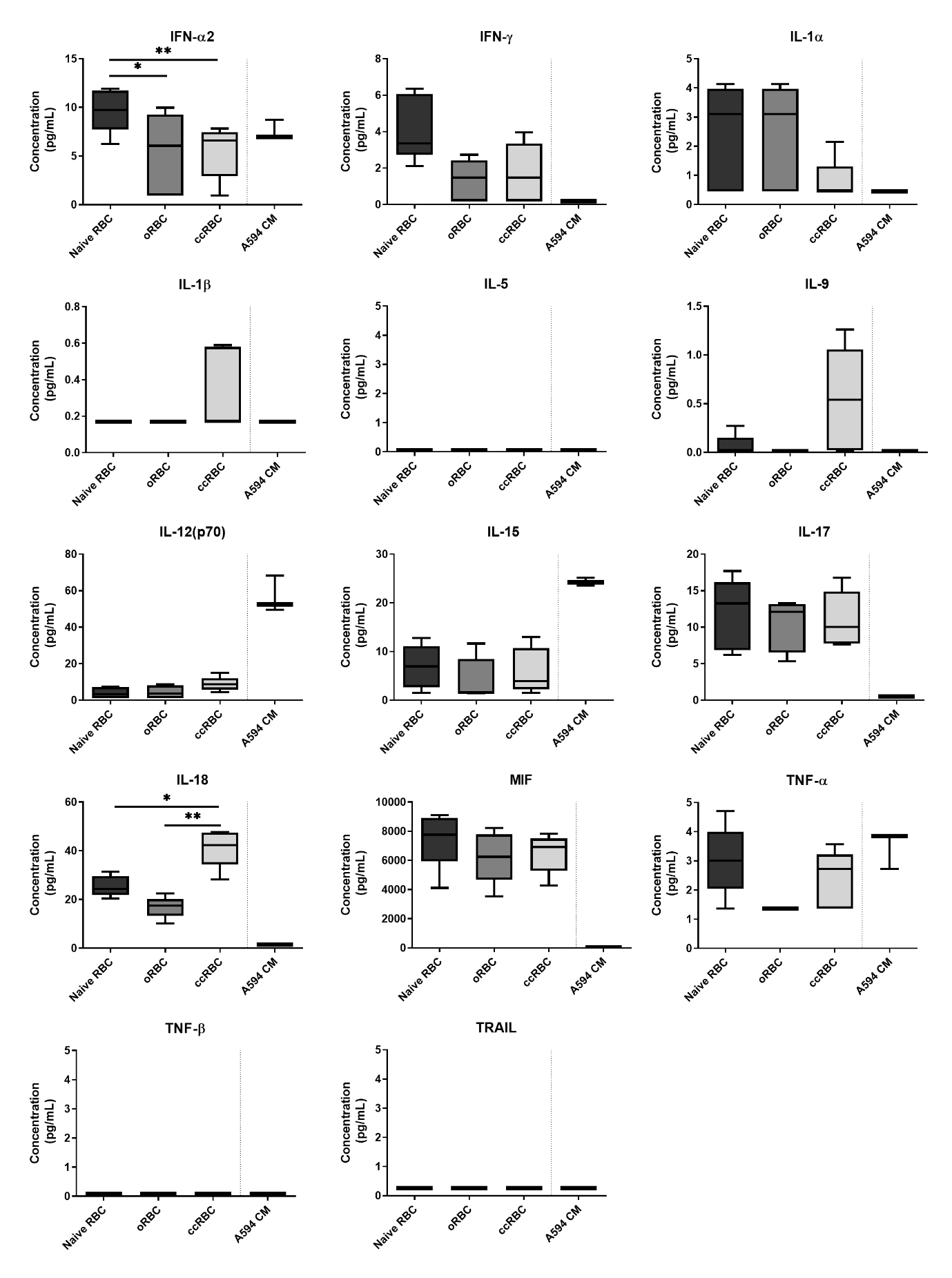


**Supplementary Figure S1. RBC acquisition of pro-inflammatory cytokines from cancer cells.** Summary of pro-inflammatory cytokines in the lysates of naïve RBCs before incubation, oRBCs and ccRBCs after incubation (3 days, cultured at a ratio of 1:100 A549:RBCs cell number), and conditioned media from A549 cells cultured alone (A549 CM) as measured by Bio-Plex. Lysate data reported as concentration (100 x 10^6^ cells/mL PBS). Data are presented as box and whisker plots with median concentration (*n* = 5). Data are statistically significantly different if *p* < 0.05 (*), *p* < 0.01 (**).


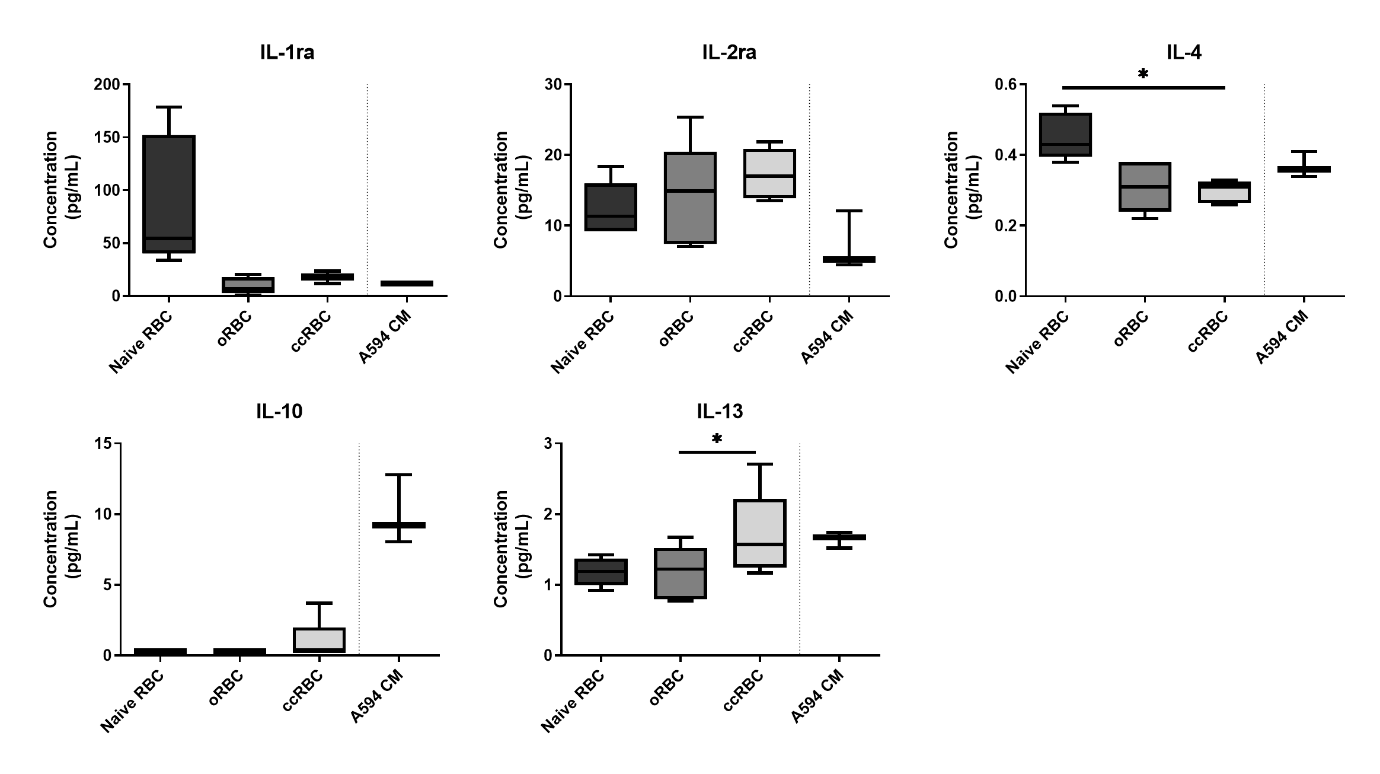


**Supplementary Figure S2. RBC acquisition of anti-inflammatory cytokines from cancer cells.** Summary of anti-inflammatory cytokines in the lysates of naïve RBCs before incubation, oRBCs and ccRBCs after incubation (3 days, cultured at a ratio of 1:100 A549:RBCs cell number), and conditioned media from A549 cells cultured alone (A549 CM) as measured by Bio-Plex. Lysate data reported as concentration (100 x 10^6^ cells/mL PBS). Data are presented as box and whisker plots with median concentration (*n* = 5). Data are statistically significantly different if *p* < 0.05 (*).


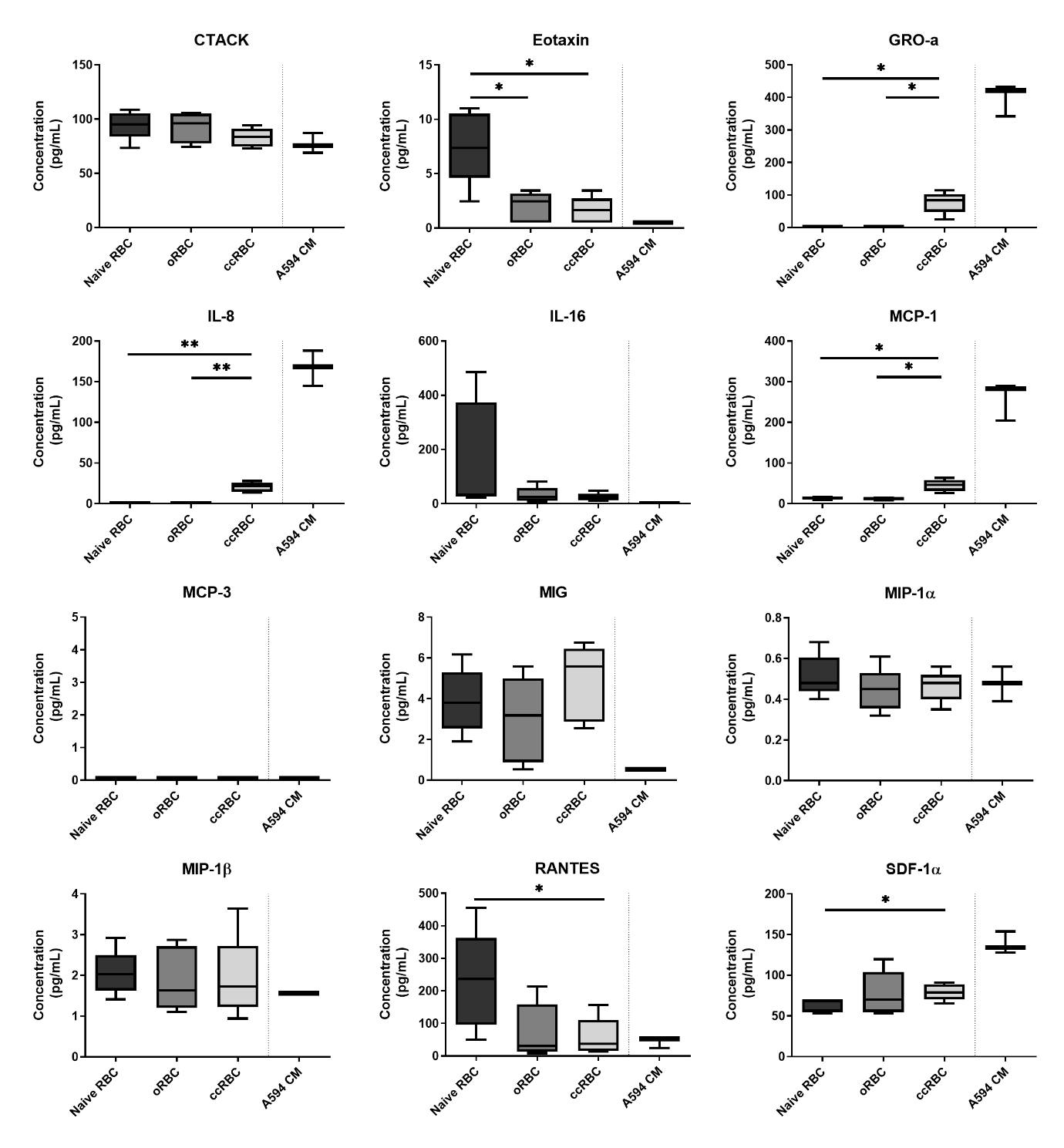


**Supplementary Figure S3. RBC acquisition of chemokines from cancer cells.** Summary of chemokines in the lysates of naïve RBCs before incubation, oRBCs and ccRBCs after incubation (3 days, cultured at a ratio of 1:100 A549:RBCs cell number), and conditioned media from A549 cells cultured alone (A549 CM) as measured by Bio-Plex. Lysate data reported as concentration (100 x 10^6^ cells/mL PBS). Data are presented as box and whisker plots with median concentration (*n* = 5). Data are statistically significantly different if *p* < 0.05 (*), *p* < 0.01 (**).


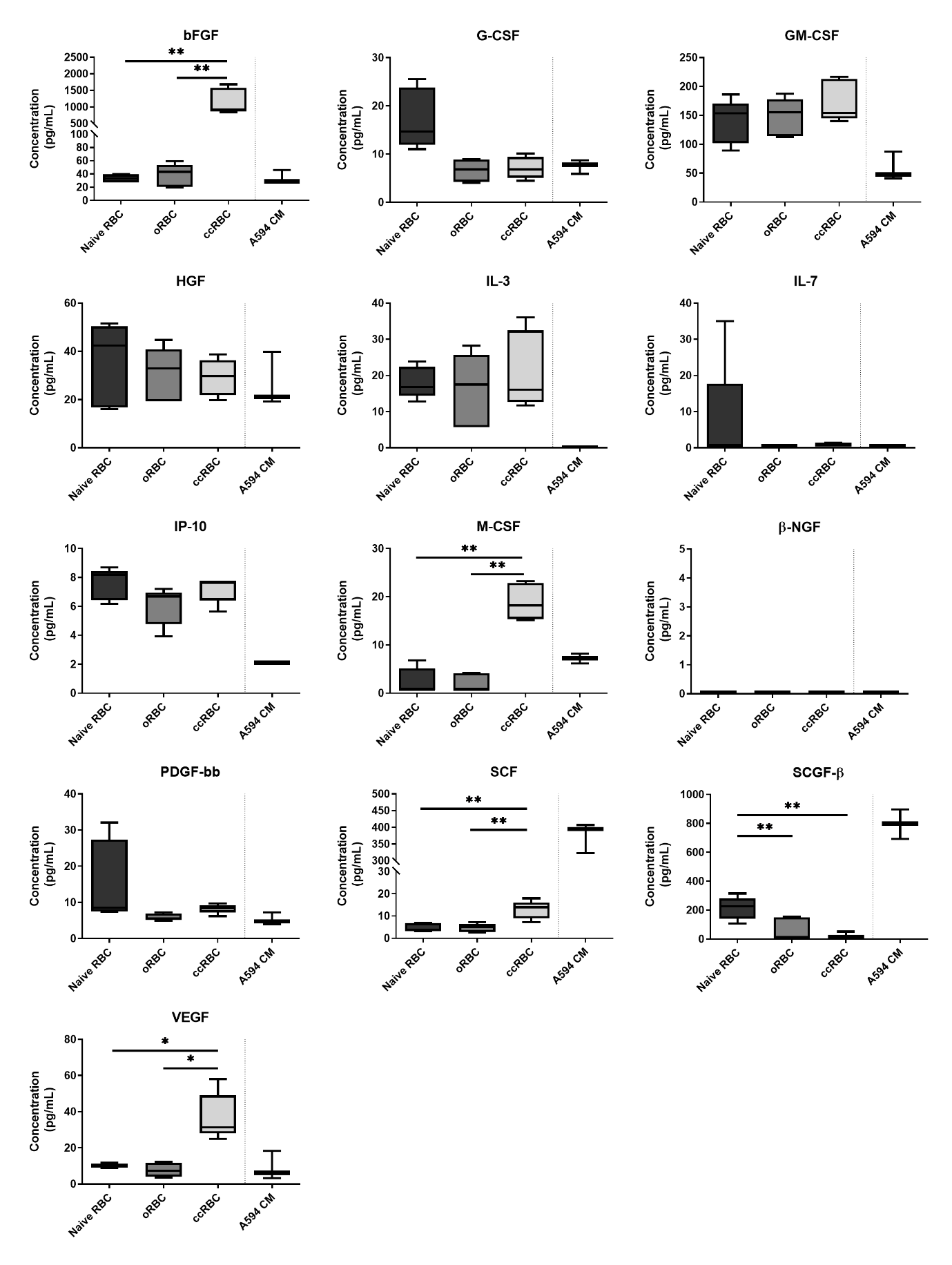


**Supplementary Figure S4. RBC acquisition of growth factors from cancer cells.** Summary of growth factors in the lysates of naïve RBCs before incubation, oRBCs and ccRBCs after incubation (3 days, cultured at a ratio of 1:100 A549:RBCs cell number), and conditioned media from A549 cells cultured alone (A549 CM) as measured by Bio-Plex. Lysate data reported as concentration (100 x 10^6^ cells/mL PBS). Data are presented as box and whisker plots with median concentration (*n* = 5). Data are statistically significantly different if *p* < 0.05 (*), *p* < 0.01 (**).


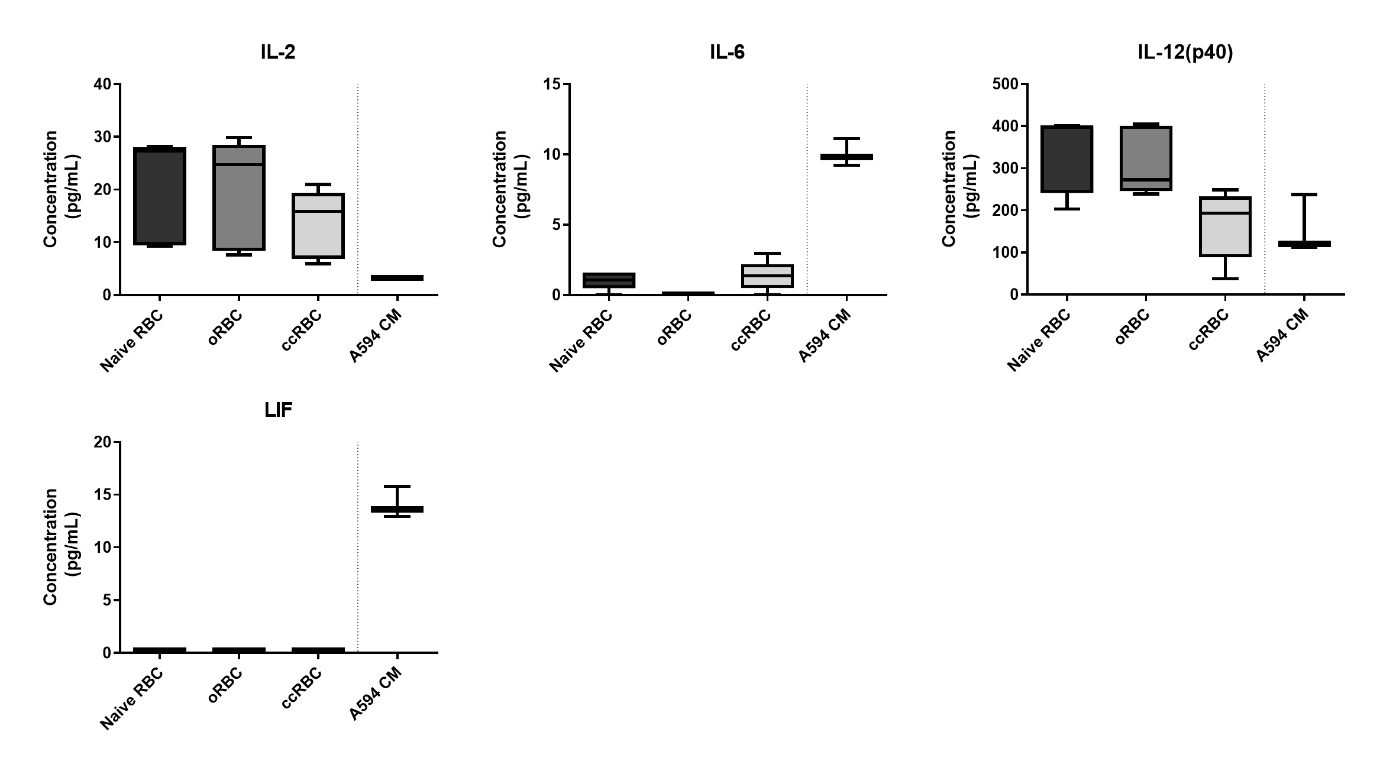


**Supplementary Figure S5. RBC acquisition of cytokines with multiple functions from cancer cells.** Summary of cytokines with multiple functions in the lysates of naïve RBCs before incubation, oRBCs and ccRBCs after incubation (3 days, cultured at a ratio of 1:100 A549:RBCs cell number), and conditioned media from A549 cells cultured alone (A549 CM) as measured by Bio-Plex. Lysate data reported as concentration (100 x 10^6^ cells/mL PBS). Data are presented as box and whisker plots with median concentration (*n* = 5). Data are statistically significantly different if *p* < 0.05 (*).


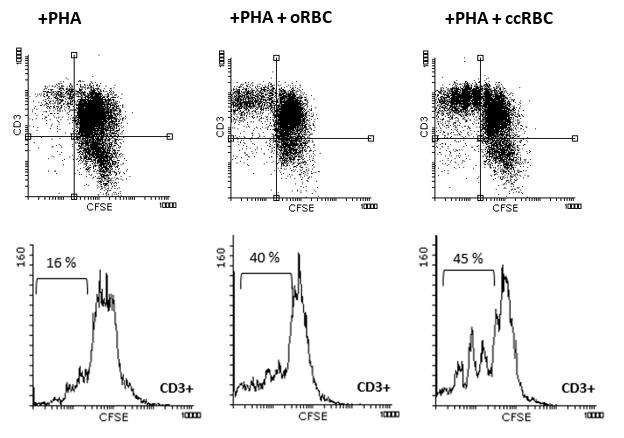


**Supplementary Figure S6. Proliferation of CD3+ peripheral blood mononuclear cells treated with red blood cells.** Histograms and dot plots show CFSE fluorescence loss in PBMCs treated with PHA-P (+ PHA), PHA-P and oRBCs (+PHA + oRBC), or PHA-P and ccRBCs (+PHA + ccRBC). The dot plots are demonstrative of the double staining for CD3+ cells and CFSE fluorescence as measured by flow cytometry. A shift in the histogram peaks to the left indicates cell proliferation over 5 days at 37 °C and 5 % CO_2_.


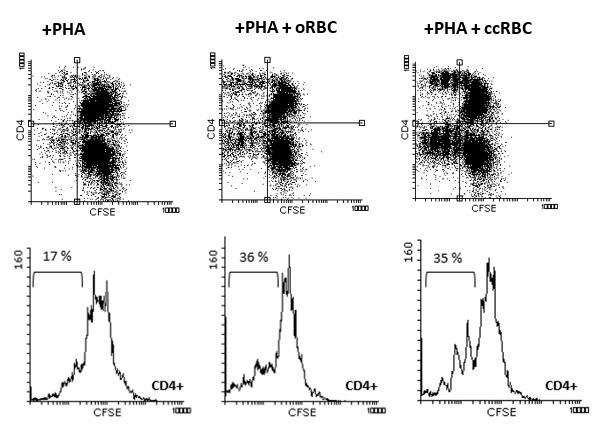


**Supplementary Figure S7. Proliferation of CD4+ peripheral blood mononuclear cells treated with red blood cells.** Histograms and dot plots show CFSE fluorescence loss in PBMCs treated with PHA-P (+ PHA), PHA-P and oRBCs (+PHA + oRBC), or PHA-P and ccRBCs (+PHA + ccRBC). The dot plots are demonstrative of the double staining for CD4+ cells and CFSE fluorescence as measured by flow cytometry. A shift in the histogram peaks to the left indicates cell proliferation over 5 days at 37 °C and 5 % CO_2_.


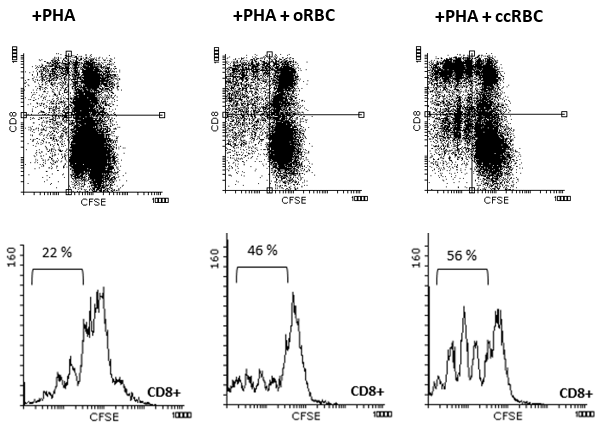


**Supplementary Figure S8. Proliferation of CD8+ peripheral blood mononuclear cells treated with red blood cells.** Histograms and dot plots show CFSE fluorescence loss in PBMCs treated with PHA-P (+ PHA), PHA-P and oRBCs (+PHA + oRBC), or PHA-P and ccRBCs (+PHA + ccRBC). The dot plots are demonstrative of the double staining for CD8+ cells and CFSE fluorescence as measured by flow cytometry. A shift in the histogram peaks to the left indicates cell proliferation over 5 days at 37 °C and 5 % CO_2_.


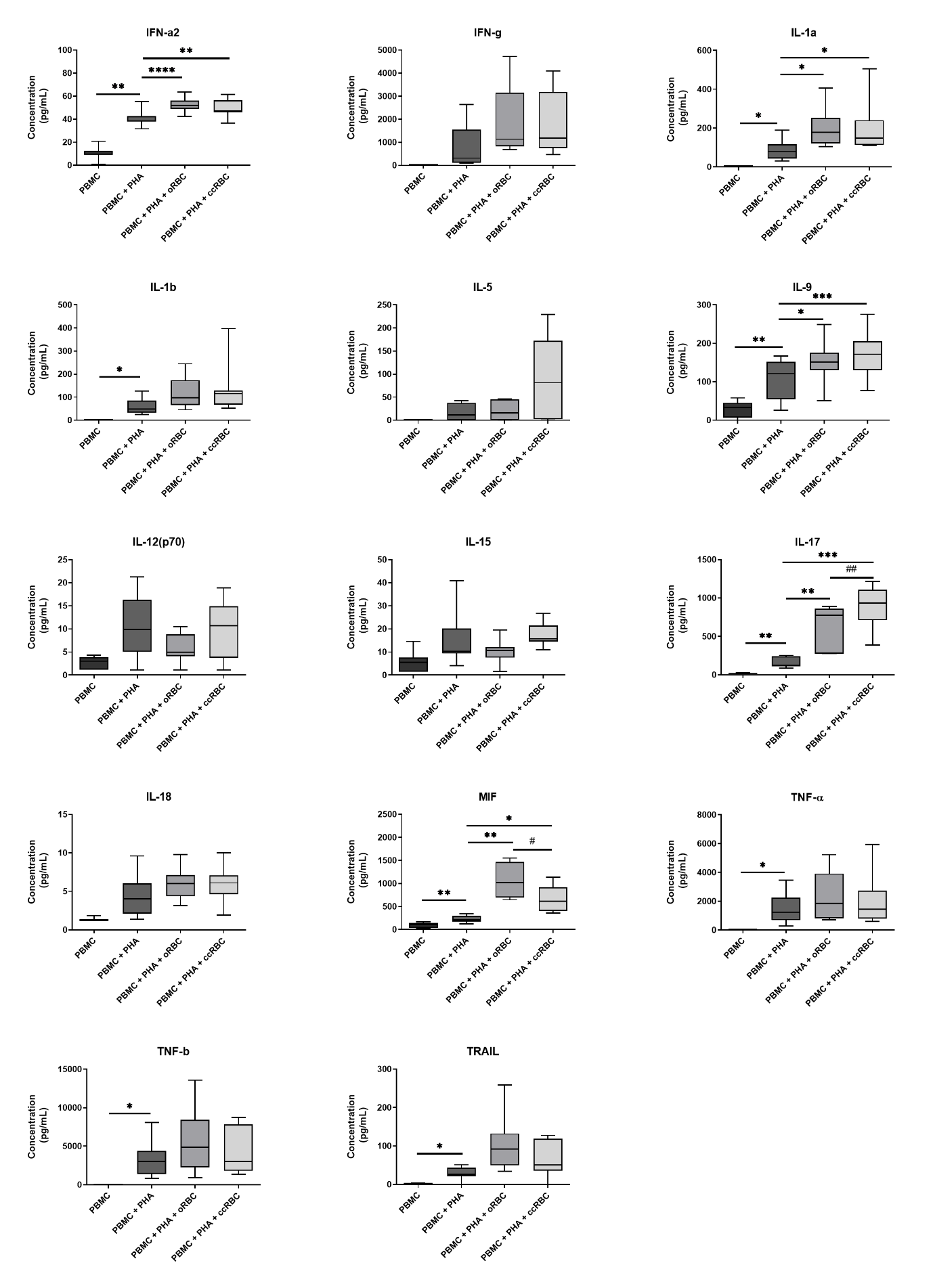


**Supplementary Figure S9. Pro-inflammatory cytokines in PBMC conditioned media.** Summary of pro-inflammatory cytokines in PBMC conditioned media from untreated PBMCs (PBMC), PBMCs treated with PHA-P (PBMC + PHA), PBMCs treated with PHA-P and oRBCs (PBMC + PHA + oRBC), and PBMCs treated with PHA-P and ccRBCs (PBMC + PHA + ccRBC) as measured by Bio-Plex. Data are presented as box and whisker plots with median concentration (*n* = 5). Data are statistically significantly different from PBMCs treated with PHA-P if *p* < 0.05 (*), *p* < 0.01 (**), *p* < 0.001 (***), or *p* < 0.0001 (****) and PBMC + PHA + oRBCs are statistically significantly different from PBMC + PHA + ccRBCs if *p*< 0.05 (#).


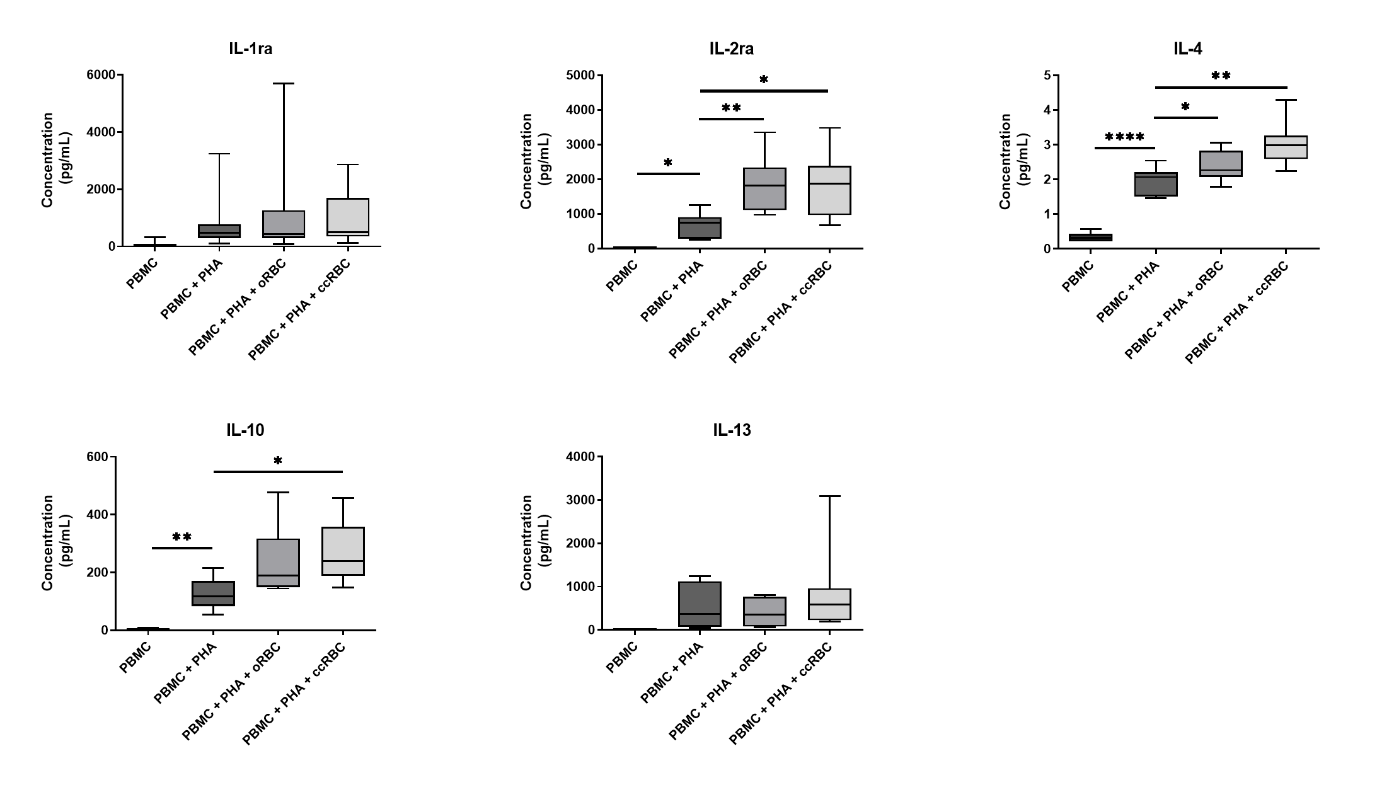


**Supplementary Figure S10. Anti-inflammatory cytokines in PBMC conditioned media.** Summary of anti-inflammatory cytokines in PBMC conditioned media from untreated PBMCs (PBMC), PBMCs treated with PHA-P (PBMC + PHA), PBMCs treated with PHA-P and oRBCs (PBMC + PHA + oRBC), and PBMCs treated with PHA-P and ccRBCs (PBMC + PHA + ccRBC) as measured by Bio-Plex. Data are presented as box and whisker plots with median concentration (*n* = 5). Data are statistically significantly different from PBMCs treated with PHA-P if *p* < 0.05 (*), *p* < 0.01 (**), *p* < 0.001 (***), or *p* < 0.0001 (****) and PBMC + PHA + oRBCs are statistically significantly different from PBMC + PHA + ccRBCs if *p*< 0.05 (#).


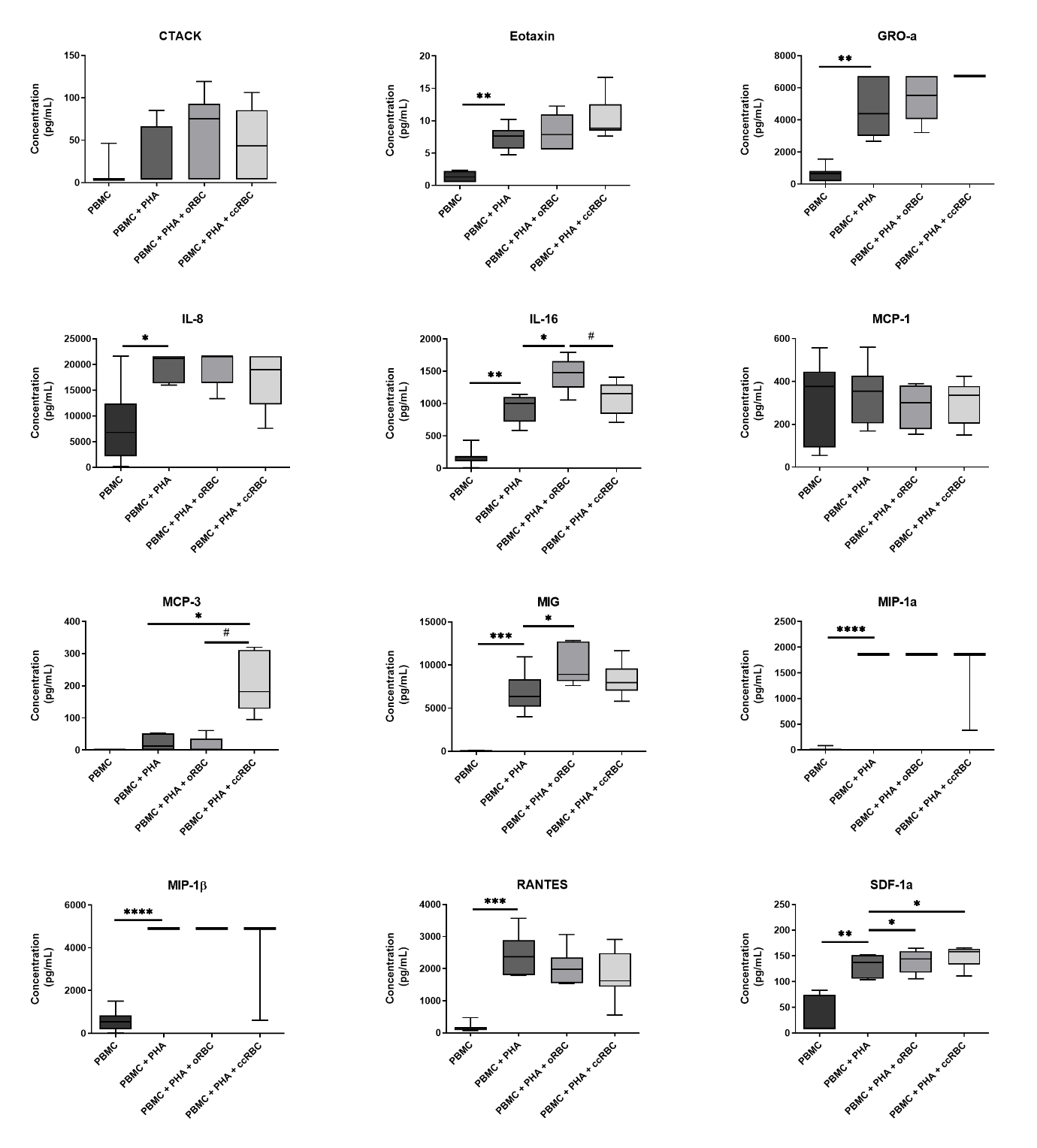


**Supplementary Figure S11. Chemokines in PBMC conditioned media.** Summary of chemokines in PBMC conditioned media from untreated PBMCs (PBMC), PBMCs treated with PHA-P (PBMC + PHA), PBMCs treated with PHA-P and oRBCs (PBMC + PHA + oRBC), and PBMCs treated with PHA-P and ccRBCs (PBMC + PHA + ccRBC) as measured by Bio-Plex. Data are presented as box and whisker plots with median concentration (*n* = 5). Data are statistically significantly different from PBMCs treated with PHA-P if *p* < 0.05 (*), *p* < 0.01 (**), *p* < 0.001 (***), or *p* < 0.0001 (****) and PBMC + PHA + oRBCs are statistically significantly different from PBMC + PHA + ccRBCs if *p*< 0.05 (#).


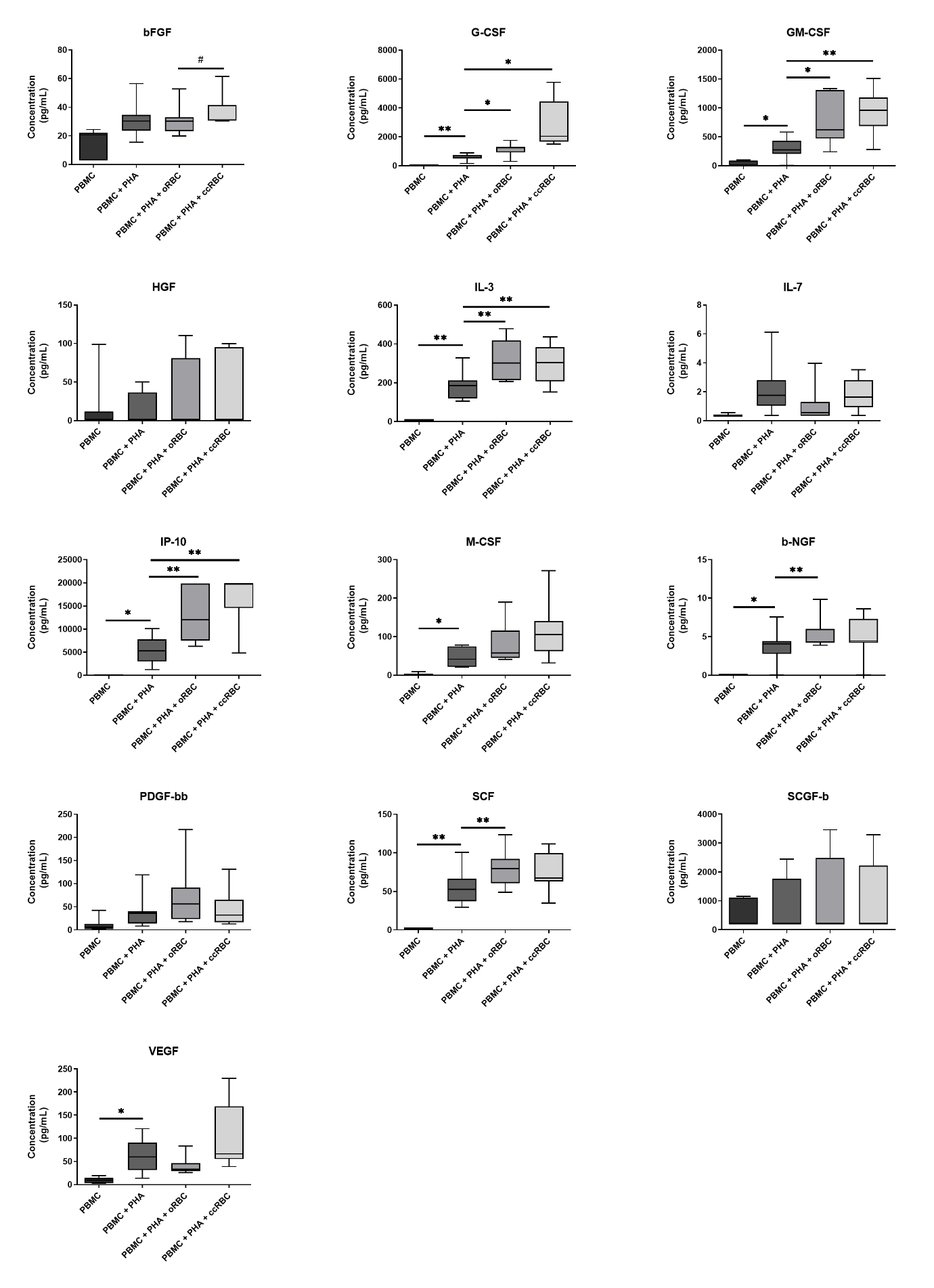


**Supplementary Figure S12. Growth factors in PBMC conditioned media.** Summary of growth factors in PBMC conditioned media from untreated PBMCs (PBMC), PBMCs treated with PHA-P (PBMC + PHA), PBMCs treated with PHA-P and oRBCs (PBMC + PHA + oRBC), and PBMCs treated with PHA-P and ccRBCs (PBMC + PHA + ccRBC) as measured by Bio-Plex. Data are presented as box and whisker plots with median concentration (*n* = 5). Data are statistically significantly different from PBMCs treated with PHA-P if *p* < 0.05 (*), *p* < 0.01 (**), *p* < 0.001 (***), or *p* < 0.0001 (****) and PBMC + PHA + oRBCs are statistically significantly different from PBMC + PHA + ccRBCs if *p*< 0.05 (#).


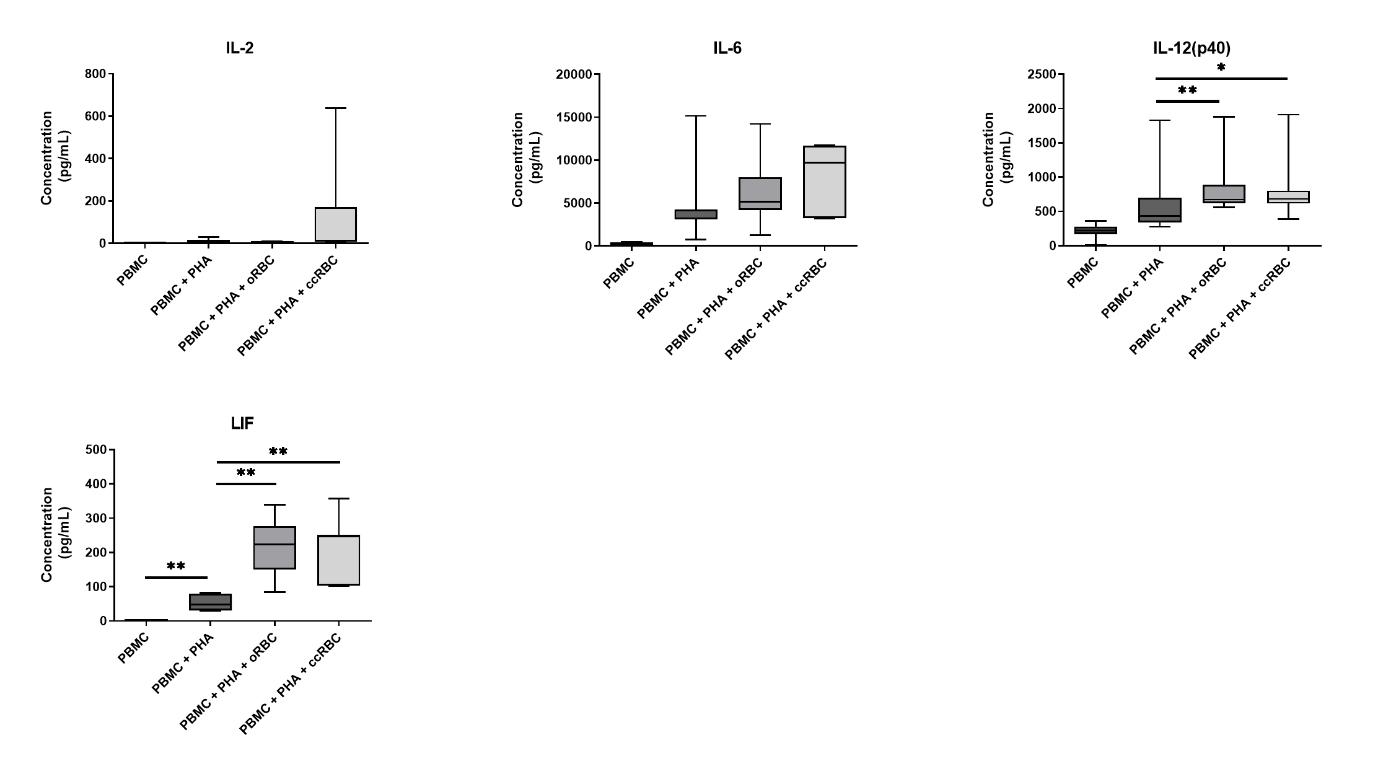


**Supplementary Figure S13.** **Cytokines with multiple functions in PBMC conditioned media.** Summary of cytokines with multiple functions in PBMC conditioned media from untreated PBMCs (PBMC), PBMCs treated with PHA-P (PBMC + PHA), PBMCs treated with PHA-P and oRBCs (PBMC + PHA + oRBC), and PBMCs treated with PHA-P and ccRBCs (PBMC + PHA + ccRBC) as measured by Bio-Plex. Data are presented as box and whisker plots with median concentration (*n* = 5). Data are statistically significantly different from PBMCs treated with PHA-P if *p* < 0.05 (*), *p* < 0.01 (**), *p* < 0.001 (***), or *p* < 0.0001 (****) and PBMC + PHA + oRBCs are statistically significantly different from PBMC + PHA + ccRBCs if *p*< 0.05 (#).
